# Long-Term Outcomes of Treosulfan- vs. Busulfan-Based Conditioning Regimen for Patients With Myelodysplastic Syndrome and Acute Myeloid Leukemia Before Hematopoietic Cell Transplantation: A Systematic Review and Meta-Analysis

**DOI:** 10.3389/fonc.2020.591363

**Published:** 2020-12-16

**Authors:** Sheng Zhu, Gang Liu, Jing Liu, Qiuying Chen, Zhiqiang Wang

**Affiliations:** ^1^ Department of Radiology, The First Affiliated Hospital of Jinan University, Guangzhou, China; ^2^ Department of Radiology, Affiliated Hospital of Xiangnan University, Chenzhou, China; ^3^ Department of Pediatrics, Affiliated Hospital of Xiangnan University, Chenzhou, China

**Keywords:** myelodysplastic syndrome, acute myeloid leukemia, allogeneic hematopoietic cell transplantation, preconditioning regimen, treosulfan, busulfan

## Abstract

**Background:**

Many studies aimed to evaluate the efficacy and safety of treosulfan-based conditioning regimens for allogeneic hematopoietic cell transplantation (allo-HCT) compared with other regimens, but different outcomes were reported across studies.

**Aim:**

To determine the long-term survival outcomes of treosulfan-based vs. busulfan-based conditioning regimens in myelodysplastic syndrome (MDS)/acute myeloid leukemia (AML) patients.

**Methods:**

PubMed, Embase, and Cochrane library were searched for studies published prior to December 6, 2019. The fixed-effects model was applied for overall survival (OS), leukemia-free survival (LFS), non-relapse mortality (NRM), acute and chronic graft versus host disease (GvHD). Relapse incidence (RI) was pooled by the use of the random-effects model.

**Results:**

Six studies were included (3,982 patients; range, 57–1,956). The pooled HR for OS favored treosulfan (HR=0.80, 95%CI: 0.71–0.90). There was no significant difference in NRM between the two regimens (HR=0.84, 95%CI=0.71–1.01). There was no significant difference in LFS between the two regimens (HR=0.98, 95%CI=0.87–1.12). Treosulfan-based regimens showed a lower risk of aGvHD (HR=0.70, 95%CI=0.59–0.82), but there was no difference for cGvHD (HR=0.94, 95%CI=0.81–1.09). There was no significant difference in RI between the two regimens (HR=0.96, 95%CI=0.71–1.31). There was no publication bias among these studies.

**Conclusion:**

The current meta-analysis determined that treosulfan-based conditioning regimens could improve the OS in patients with MDS and AML, with lower acute graft-versus-host disease incidence, compared with busulfan-based regimens.

## ntroduction

Myelodysplastic syndrome (MDS) is a heterogeneous group of clonal disorders characterized by ineffective hematopoiesis leading to peripheral blood cytopenias and increased risk of transformation to acute myeloid leukemia (1–3). The incidence of MDS in the USA is 4.5 per 100,000 people/year in men and 2.7 per 100,000 people/year in women (4). Acute myeloid leukemia (AML) is a collection of heterogeneous hematopoietic stem cell disorders characterized by incomplete maturation of blood cells and reduced production of normal hematopoietic elements (5, 6). There are an estimated 18,860 new cases of AML and 10,460 deaths from AML in the United States each year (7).

Allogeneic hematopoietic cell transplantation (HCT) offers curative therapy for many patients with MDS or AML. Allo-HSCT is recommend for MDS of intermediate-2- and high-risk as soon as possible, according to The National Comprehensive Cancer Network (NCCN) guidelines (1, 6). Nevertheless, relapse and graft versus host disease (GvHD) are the two main causes of treatment failure (1, 6). Thus the choice of the conditioning regimen is crucial.

Conditioning regimens play important roles in the success of allo-HSCT, and the choice of a regiment depends upon age, disease risk, ECOG status, and remission status at the time of transplantation (8, 9). Conventional myeloablative conditioning regimens (MAC) containing busulfan are among the most widely used for MDS and AML, but they have shortcomings such as toxicity, veno-occlusive diseases, and mortality (8–10). Treosulfan is a recent myelotoxic and immunosuppressive prodrug that does not require enzymatic activation (11, 12). Treosfulfan has strong activity against AML cells (13–15) and has strong immunosuppressive effects and low release of inflammatory cytokines (16). Those characteristics favor the engraftment of the transplanted cells and limit the risk of GvHD (16). Treosulfan-based regimens are considered as effective as conventional MAC, but with lower toxicity and transplant-related mortality (8, 9).

Many studies aimed to evaluate the efficacy and safety of treosulfan-based conditioning regimens compared with other regimens, but different outcomes were reported across studies. No meta-analysis has been published yet as far as we know. Therefore, the purpose of this meta-analysis was to determine the long-term survival outcomes of treosulfan-based conditioning regimens in MDS/AML patients.

## Methods

### Literature Search

This meta-analysis was performed in accordance with the Preferred Reporting Items for Systematic Reviews and Meta-Analyses (PRISMA) reporting guidelines. Since no original clinical raw data was collected or utilized, ethical approval was not requested for this meta-analysis.

The study eligibility criteria were: 1) patients: AML/MDS patients older than 18 years (either mentioned in patient eligibility or the minimum age in baseline characteristics was greater than 18); 2) interventions: treosulfan as conditioning regimen before hematopoietic cell transplantation; 3) comparison: busulfan or other conditioning regimens; 4) study types: randomized controlled trials (RCTs), and prospective or retrospective observational cohort studies; and 5) full text published in English. Three recognized electronic databases, PubMed, Embase, and Cochrane library, were searched for studies published prior to December 6, 2019, using the MeSH terms of “Myelodysplastic Syndrome”, “Acute Myeloid Leukemia”, and “treosulfan” combined with relevant key words were used.

### Data Extraction and Quality Assessment

Potentially relevant publications were screened and evaluated by two reviewers double-blindly, with a third reviewer resolving any disagreement. A structured data collection sheet was developed. Two researchers independently extracted the data, including authors, year of publication, country, study design, sample size, age, percentage of males, disease and transplantation characteristics, conditioning regimens characteristics, overall survival (OS), leukemia-free survival (LFS), non-relapse mortality (NRM), acute GvHD (aGvHD), chronic GvHD (cGvHD), and relapse incidence (RI). RCTs were evaluated according to the Cochrane risk bias tool (4). Observational studies were evaluated according to the Newcastle-Ottawa scale (NOS) (17). The NOS assigns a maximum of 9 points for the selection of the control group (4 points), group comparability (two points), and exposures and outcomes (three points).

### Statistical Analysis

All analyses were performed using the STATA MP 14.0 software (StataCorp, College Station, Texas, USA). Hazard ratios (HRs) with 95% confidence intervals (CIs) were collected and combined for statistical analysis. Statistical heterogeneity among studies was calculated using the Cochran’s Q test and I^2^ index, P<0.10 in the Cochran’s Q test and an I^2^ index >50% indicated high heterogeneity. The random-effects model was used when high heterogeneity was present among studies; otherwise, the fixed-effects model was applied. P-values <0.05 were considered statistically different. Potential publication bias was assessed by funnel plots, Egger’s test, and Begg test.

## Results

### Study Selection

**Figure 1:**
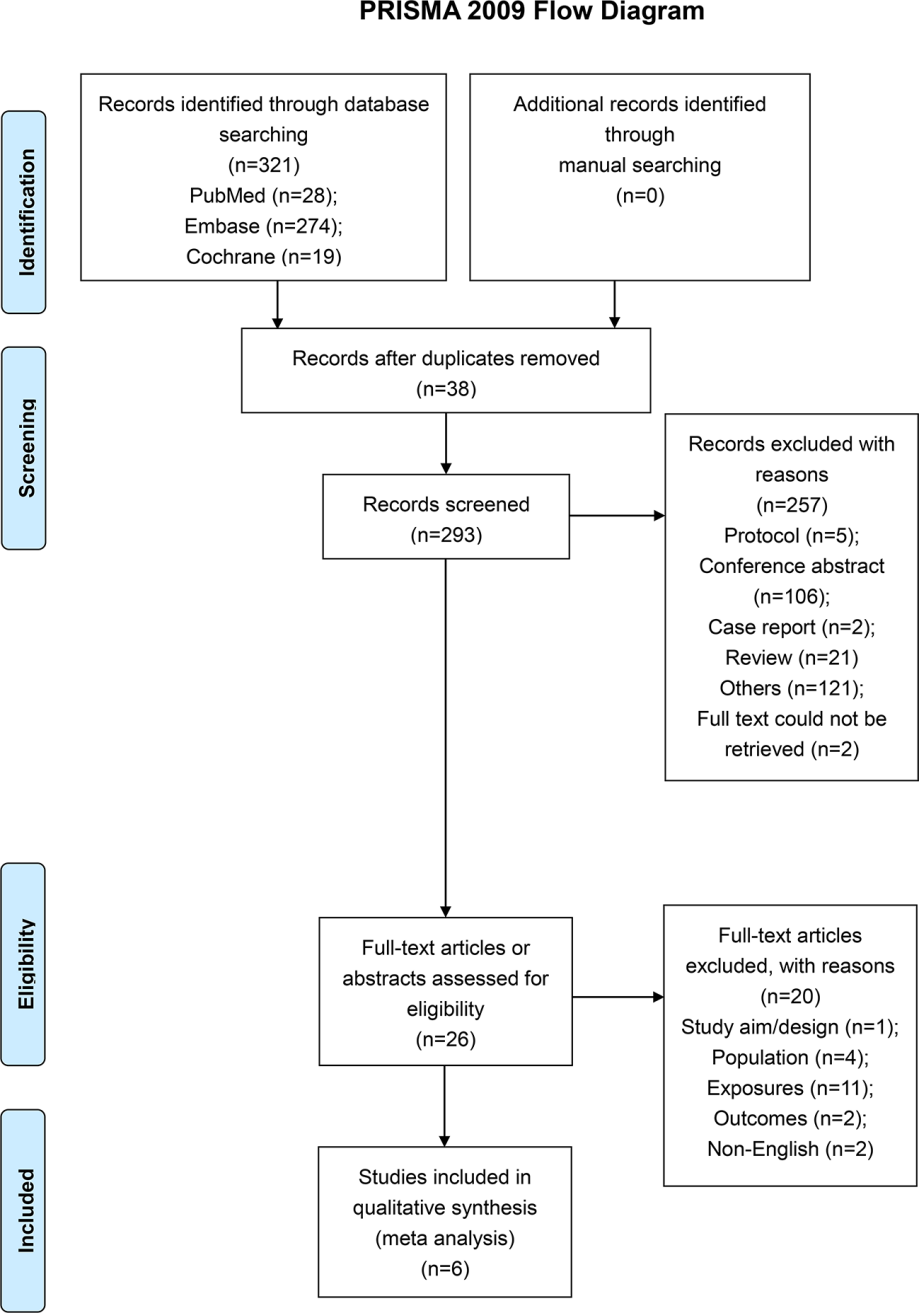
Study flowchart.

A total of 321 articles were first identified, 38 duplicates were removed, and 293 papers were screened. Twenty-six full-text were assessed for eligibility. Twenty studies were excluded because of study aim/design (n=1), population (n=4), exposures (n=11), outcomes (n=2), and non-English (n=2) (**Figure 1** and **Supplementary Table 1**). Finally, six papers were included (**Figure 1**). Those six studies (18–23) included 3,982 patients (range, 57-1956) (**Table 1**). There were five observational studies (19–23) and one randomized trial (18). **Tables 2** and **3** presents quality assessment.

**Table 1:** General characteristics of included studies.

Study	Country/Race	Design	Intervention	No. of patients	Age (years, mean or median)	Gender	Disease		Transplantation characteristics			Conditioning regimens		Outcome of interest
						Male (%)	With AML	With MDS	Graft source	Donor’s characteristics	Fludarabine	Treosulfan	Busulfan	
Beelen (18)	France, Germany, Hungary, Italy, and Poland	Multicentre, open-label, randomised, non-inferiority trial	FT	220	60.0 (55.0–65.0)	130/220 (59%)	155/220 (71%)	65/220 (30%)	214 (97%) PBSC	52(24%) MRD	IV 30 mg/m^2^	14 g/m^2^, 10 g/m^2^		OS, EFS, NRM, TRM, Cumulative relapse or progression incidence,aGvHD,cGvHD,
			FB	240	61.0 (56.5–64.0)	149/240 (62%)	138/240 (58%)	102/240 (43%)	235 (98%) PBSC	59 (25%) MRD	IV 30 mg/m^2^		0.8 mg/kg	
Saraceni (19)	EBMT; (Italy, France, Germany, USA, Israel)	Retrospective cohort	FT	113	58 (47-64)	66/113 (58%)	113 (100%)		109 (96%) PBSC	56 (49%) MSD		30, 36, or 42 mg/m^2^		OS, NRM, LFS, RI, aGvHD,cGvHD, GRFS
			FLAMSA	631	51.5 (41.9-59.9)	336/631 (53%)	631 (100%)		616 (98%) PBSC	252 (40%) MSD			6.4, 9.6, 12.8 mg/kg	
Sheth (20)	ALWP/EBMT multicenter registry; (UK, France, Israel, Finland, Germany)	Retrospective cohort	Treo/Flu	281	57 (40.2 – 64.9)	148/281 (52.67%)	281 (100%)		245 (87.19%) PBSC	90 (32.03%) MSD	≥ 150 mg/m^2^ total dose	>30 g/m^2^total dose		OS, NRM, LFS, RI, aGvHD,cGvHD
			FLAMSA/Bu	145	59 (40.7 – 65)	82/145 (56.55%)	145 (100%)		138 (95.17%) PBSC	33 (22.76%) MSD	≥ 150 mg/m^2^ total dose		6 mg/kg	
Shimoni (21)	ALWP of the EBMT; (Israel, France, USA, Algeria, Germany, UK)	Retrospective cohort	FT12	168	60 (21-73)	52%	168 (100%)		95% PBSC	33% MSD		30 to 36g/m^2^		OS, NRM, LFS, RI, aGvHD,cGvHD
			FT14	403	57 (19-73)	50%	403 (100%)		90% PBSC	36% MSD		a total dose of 42g/m^2^		
			FB4	1533	48 (18-74)	55%	1533 (100%)		85% PBSC	62% MSD			a total dose of 12.8 mg/kg	
Sakellari (23)	Greece	Retrospective cohort	FluTreo	31	55 (25-65)		25 (81%)	6 (19%)	100% PBSC	12 (39%) MSD	150 mg/m^2^	42 g/m^2^		OS, DFS, TRM, Relapse mortality
			FluBuATG	26	56 (26-63)		21 (81%)	5 (19%)	100% PBSC	22 (85%) MSD	150 to 180mg/m^2^		6.4 mg/kg	
Shimoni (22)	Israel	Prospective cohort	FT	85	59 (25-76)	51/85 (60%)	50 (59%)	35 (41%)		38 (45%) MSD	30 mg/m^2^	12 g/m^2^		OS, NRM, RI
			FB2	106	60 (29-75)	61 (58%)	90 (85%)	16 (15%)		47 (44%) MSD	30 mg/m^2^		3.2 mg/kg	

F/Flu, fludarabine; B/Bu, busulfan; T/Treo, treosulfan; EBMT, European Society for Blood and Marrow Transplantation; ALWP, Acute Leukemia Working Party; FLAMSA, fludarabine, intermediate-dose Ara-C, amsacrine, total body irradiation/busulfan, cyclophosphamide; LFS, leukemia-free survival; OS, overall survival; GRFS, GVHD-free relapse-free survival; AML, acute myeloid leukemia; MDS, myelodysplastic syndrome; FT, fludarabine-treosulfan; FB, busulfan plus fludarabine; TBF, thiotepa-busulfan-fludarabine; ATG, antithymocyte globulin; RI, relapse incidence; NRM, non-relapse mortality incidence; DFS, disease-free survival; TRM, transplantation-related mortality; aGvHD, acute graft-versus-host disease; cGvHD, chronic graft-versus-host disease; EFS, event-free survival; PBSC, peripheral blood stem cells; MRD, matched related donor; MSD, matched sibling donor.

**Table 2:** Quality assessment of included studies based on Newcastle-Ottawa scale (NOS).

Study	Representativeness of the exposed cohort	Selection of the non exposed cohort	Ascertainment of exposure	Demonstration that the outcome of interest was not present at the start of the study	Comparability of cohorts on the basis of the design or analysis	Assessment of outcome	Was follow-up long enough for outcomes to occur	Adequacy of follow up of cohorts	Total quality scores
Saraceni (19)	☆	☆	☆		☆☆	☆	☆	☆	8
Sheth (20)	☆	☆	☆		☆☆	☆	☆	☆	8
Shimoni (21)	☆	☆	☆		☆☆	☆	☆	☆	8
Sakellari (23)	☆	☆	☆		☆☆	☆	☆	☆	8
Shimoni (22)	☆	☆	☆	☆	☆☆	☆	☆		8

☆ and ☆☆ indicates the quality score is 1 and 2, respectively.

**Table 3:** Quality assessment of included studies based on the Cochrane tool.

Study	Random sequence generation (selection bias)	Allocation concealment (selection bias)	Blinding of participants and personnel (performance bias)	Blinding of outcome assessment (detection bias)	Incomplete outcome data (attrition bias)	Selective reporting (reporting bias)	Other bias
Beelen (18)	Low	Low	Low	Low	Low	Low	Unclear

### Overall Survival

**Figure 2:**
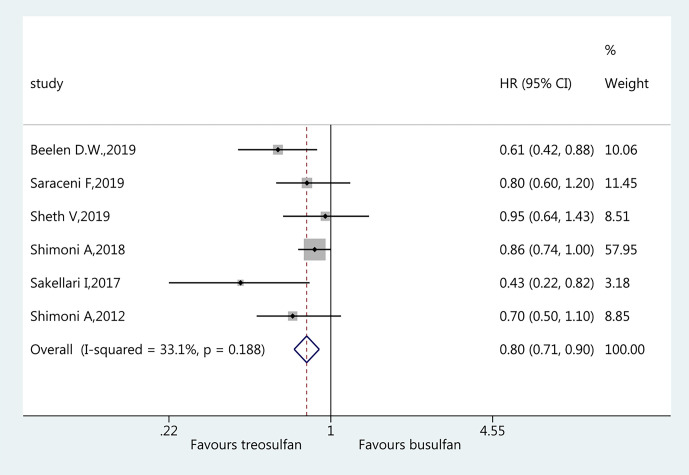
Forest plot of overall survival.

All six studies (18–23) were included for the assessment of OS. The pooled HR favored treosulfan (HR=0.80, 95%CI: 0.71–0.90). No heterogeneity was observed (I^2 ^= 33.1%, P=0.188) (**Figure 2**).

### Non-Relapse Mortality

**Figure 3:**
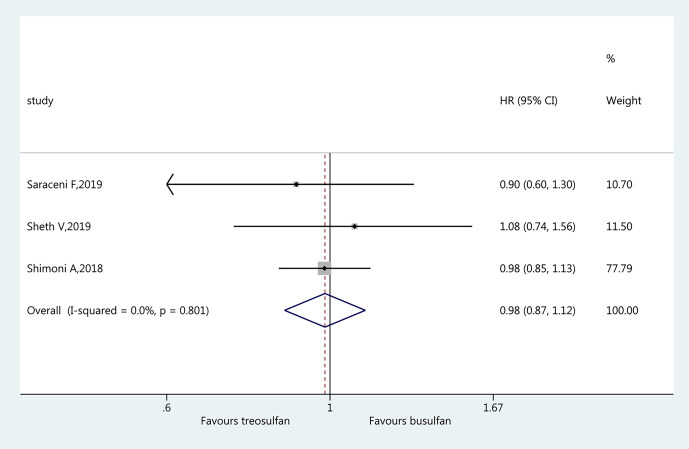
Forest plot of non-relapse mortality.

Five studies (18–22) were included in the assessment of NRM. There was no significant difference in NRM between the two regimens (HR=0.84, 95%CI=0.71–1.01). No heterogeneity was observed (I^2 ^= 16.5%, P=0.309) (**Figure 3**).

### Leukemia-Free Survival

**Figure 4:**
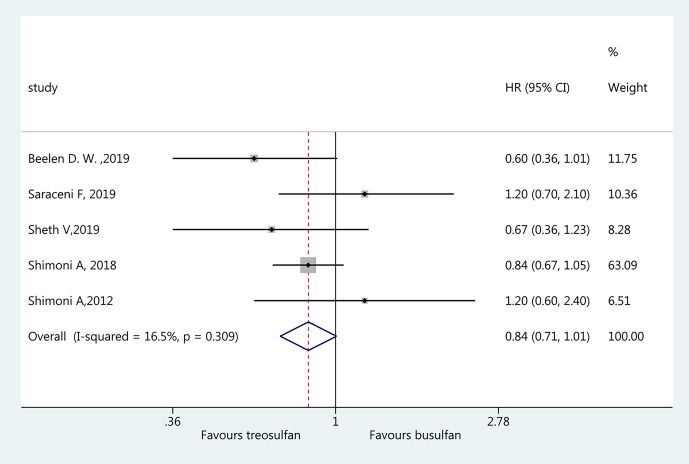
Forest plot of leukemia-free survival.

Three studies (19–21) were included in the assessment of LFS. There was no significant difference in LFS between the two regimens (HR=0.98, 95%CI=0.87–1.12). No heterogeneity was observed (I^2 ^= 0%, P=0.801) (**Figure 4**).

### Graft Versus Host Disease

**Figure 5:**
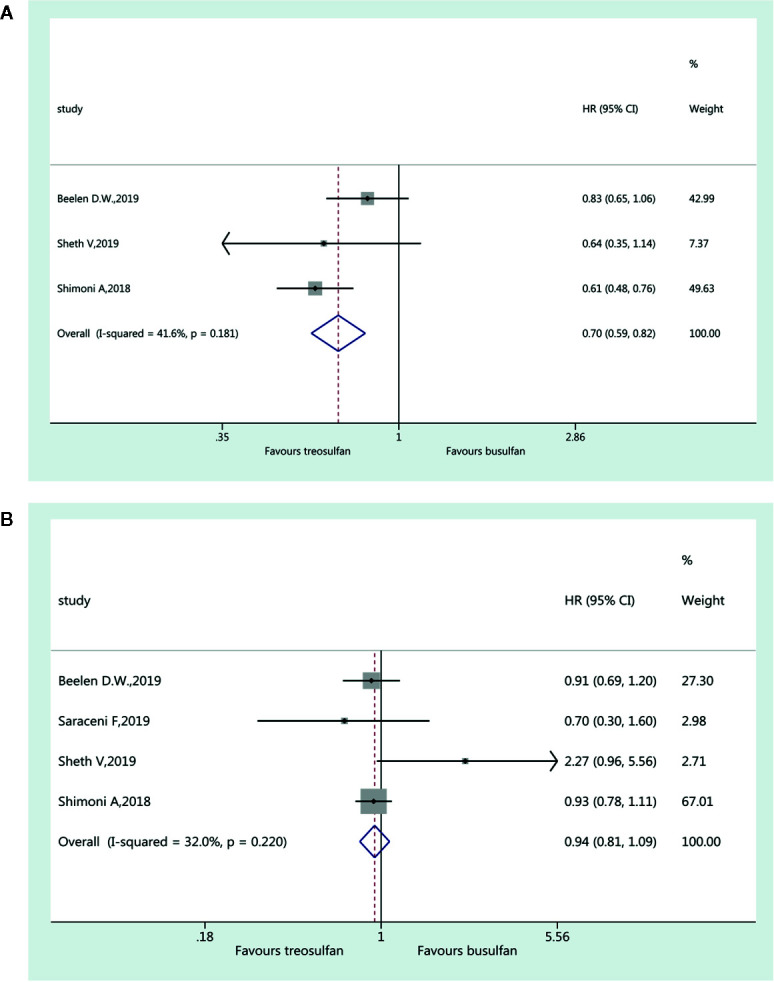
Forest plot of acute **(A)** and chronic **(B)** graft versus host disease.

Three studies (19–21) were included for the assessment of aGvHD and favored treosulfan-based regimens (HR=0.70, 95%CI=0.59–0.82). No heterogeneity was observed (I^2 ^= 41.6%, P=0.181) (**Figure 5A**). Four studies (18–21) were included in the assessment of cGvHD. There was no significant difference in cGvHD between the two regimens (HR=0.94, 95%CI=0.81–1.09). No heterogeneity was observed (I^2 ^= 32.0%, P=0.220) (**Figure 5B**).

### Relapse Incidence

**Figure 6:**
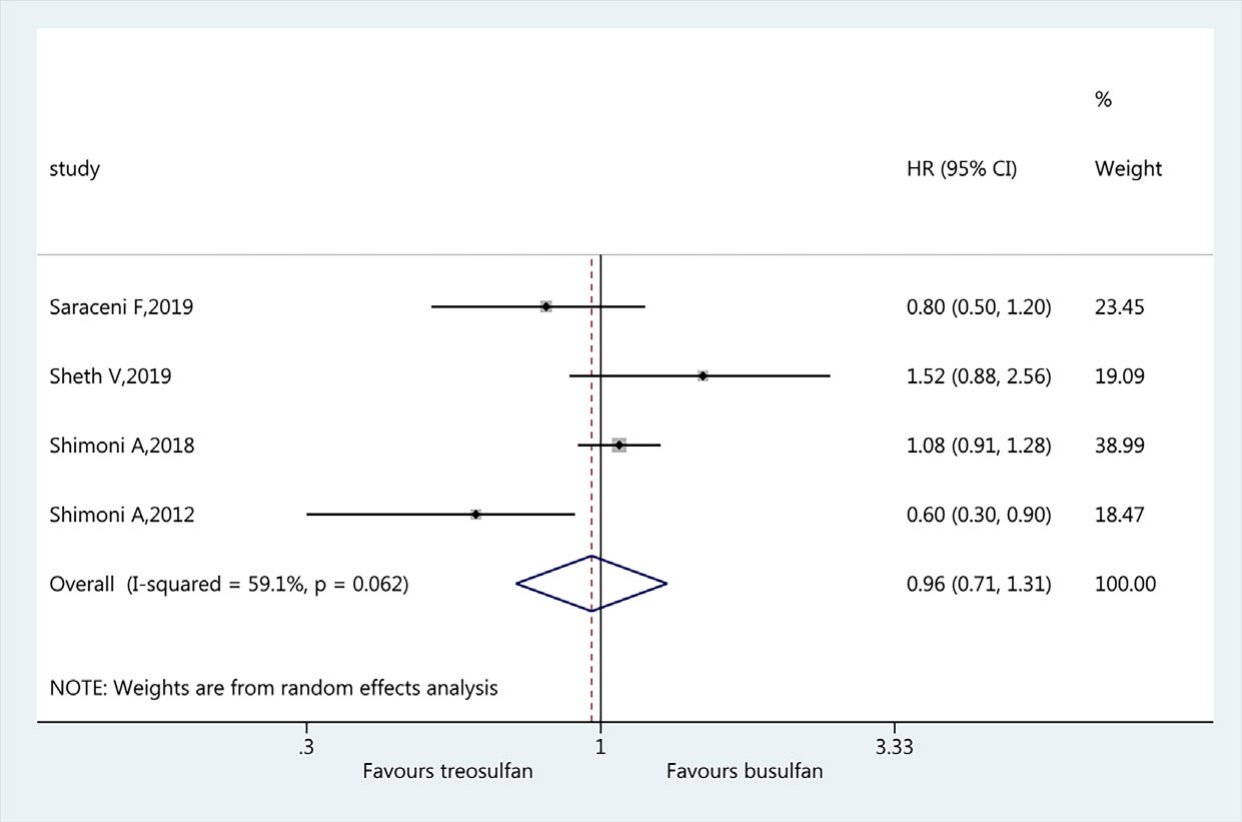
Forest plot of relapse incidence.

Four studies (19–22) were included in the assessment of RI. There was no significant difference in RI between the two regimens (HR=0.96, 95%CI=0.71–1.31). Heterogeneity was observed (I^2 ^= 59.1%, P=0.062) (**Figure 6**).

### Assessment of Publication Bias

The results of the Begg’s test showed P=0.260 and P=0.806, and the Egger’s test showed P=0.125 and P=0.868 for OS and NRM, respectively, indicating that there was no publication bias among these studies (**Supplementary Figures 1**, **2**).

## Discussion

Many studies aimed to evaluate the efficacy and safety of treosulfan-based conditioning regimens for allo-HCT compared with other regimens, but different outcomes were reported across studies. Therefore, this meta-analysis aimed to determine the long-term survival outcomes of treosulfan-based conditioning regimens in MDS/AML patients. The results suggest that treosulfan-based conditioning regimens improve the overall survival in patients with MDS and AML, with lower acute graft-versus-host disease incidence. It adds to the conclusion of the only randomized controlled trial included here (Beelen’s (18)), which showed that treosulfan-based regimens were non-inferior to busulfan when combined with fludarabine. The other five studies that could be included in the meta-analysis were retrospective studies, highlighting the need for additional well-designed randomized controlled trials to compared treosulfan- vs. busulfan-based conditioning regimens in patients MDS/AML, in addition to the results of the meta-analysis itself. This is also the main limitation of the present meta-analysis, and the interpretation of the results should be made with caution.

Conventional MAC containing busulfan are among the most widely used for MDS and AML, but they have shortcomings such as toxicity and transplantation-related mortality (8–10). Treosulfan-based regimens are considered as effective as conventional MAC, but with lower toxicity and transplant-related mortality (8, 9). Accordingly, the present study showed that OS was better with treosulfan-based regimens, and the incidence of aGvHD was lower. Indeed, aGvHD is a severe complication of allo-HSCT and can result in significant morbidity and mortality (24, 25). On the other hand, the occurrence of cGvHD was similar between the two regimens. The traditional busulfan-based regimens are still those being recommended by guidelines (1, 6, 26), but recent data support the use of treosulfan-based regimens because of the suggested better toxicity profile and similar efficacy (8, 9). This similar efficacy has been confirmed in the present meta-analysis, but safety was not directly assessed. Nevertheless, no heterogeneity or publication bias was observed for the main outcomes, suggesting a goods reliability of the results. A number of clinical trials are currently underway and should provide additional insights soon. In the meantime, Nagler et al. (27) reported a low occurrence of veno-occlusive diseases (2%) and deaths (0.4%) with treosulfan, but the lack of a comparator group precluded an actual analysis of safety. Nemecek et al. (28), in a phase II trial without a comparator, showed that the frequencies of aGvHD and cGvHD were 22% and 40%, respectively. Again, such favorable results were also observed in previous studies (29–37).

In addition, NRM, LFS, and RI, which are leukemia-related indicators, were not associated with the use of treosulfan-based regimens. This is supported by a recent report by Nagler et al. (27), who showed that treosulfan-based regimes had a favorable long-term OS and NRM profile, but they did not have a comparator group. A phase II clinical trial, without comparator, showed high OS and LFS and low NRM, also suggesting the clinical benefits of treosulfan in preconditioning regimens (28). Nemecek et al. (38) showed that NRM was 4% at 100 days and 8% at 2 years in patients who received treosulfan/fludarabine preconditioning. A number of non-comparative studies also support those conclusions in various hematologic malignancies, including AML and MDS (29–37). The main reason why those studies could not be included in the present meta-analysis is that they had no comparator group. They do show interesting and even promising results, and the reported NRM, OS, and LFS are similar to those of the studies included in the present meta-analysis, but without a comparator, the exact impact and benefits of treosulfan cannot be determined since its effects might vary among study populations, countries, and supporting treatments, among others.

This meta-analysis has limitations. Only six studies could be included, introducing bias and limiting the generalizability of the results. There was wide variability in the exact regimens and patient characteristics among the studies, leading to significant heterogeneity. Of note, there was only one randomized controlled trial, and the other included studies were retrospective. Such trials are needed to determine the exact benefits of treosulfan-bases regimens for allo-GSCT for patients with MDS or AML.

The present meta-analysis determined that treosulfan-based conditioning regimens improve the OS in patients with MDS and AML, with lower aGvHD incidence.

## Data Availability Statement

The original contributions presented in the study are included in the article/**Supplementary Material**. Further inquiries can be directed to the corresponding authors.

## Author Contributions

JL conceived and designed the study. SZ and GL performed the literature search, data extraction, quality assessment of the included studies. QC and ZW performed the statistical analysis. JL wrote the paper. JL, SZ, GL, QC, and ZW reviewed and edited the manuscript. All authors contributed to the article and approved the submitted version.

## Funding

This work was supported by The National Natural Science Foundation of China [81571664, 81871323, 81801665]; The National Natural Science Foundation of Guangdong Province [2018B030311024]; The China Postdoctoral Science Foundation [2016M600145]; the Tumor Interventional Clinical Medical Technology Demonstration Base of Hunan Province [2017SK4010]; and the Science and Technology Planning Project of Chenzhou City, Hunan Province [jsyf2017020].

## Conflict of Interest

The authors declare that the research was conducted in the absence of any commercial or financial relationships that could be construed as a potential conflict of interest.

## Supplementary Material

Supplementary Figure 1
**(A)** Sensitivity analysis of overall survival. **(B)** Sensitivity analysis of non-relapse mortality

Supplementary Figure 2
**(A)** Funnel plot of overall survival. **(B)** Funnel plot of non-relapse mortality.
