# Supplementary figures and images for: Long-Term Outcomes of Treosulfan- vs. Busulfan-Based Conditioning Regimen for Patients With Myelodysplastic Syndrome and Acute Myeloid Leukemia Before Hematopoietic Cell Transplantation: A Systematic Review and Meta-Analysis
Source: Front Oncol. 2020 Dec 16;10:591363. doi: 10.3389/fonc.2020.591363 (PMC7793760; doi:10.3389/fonc.2020.591363)

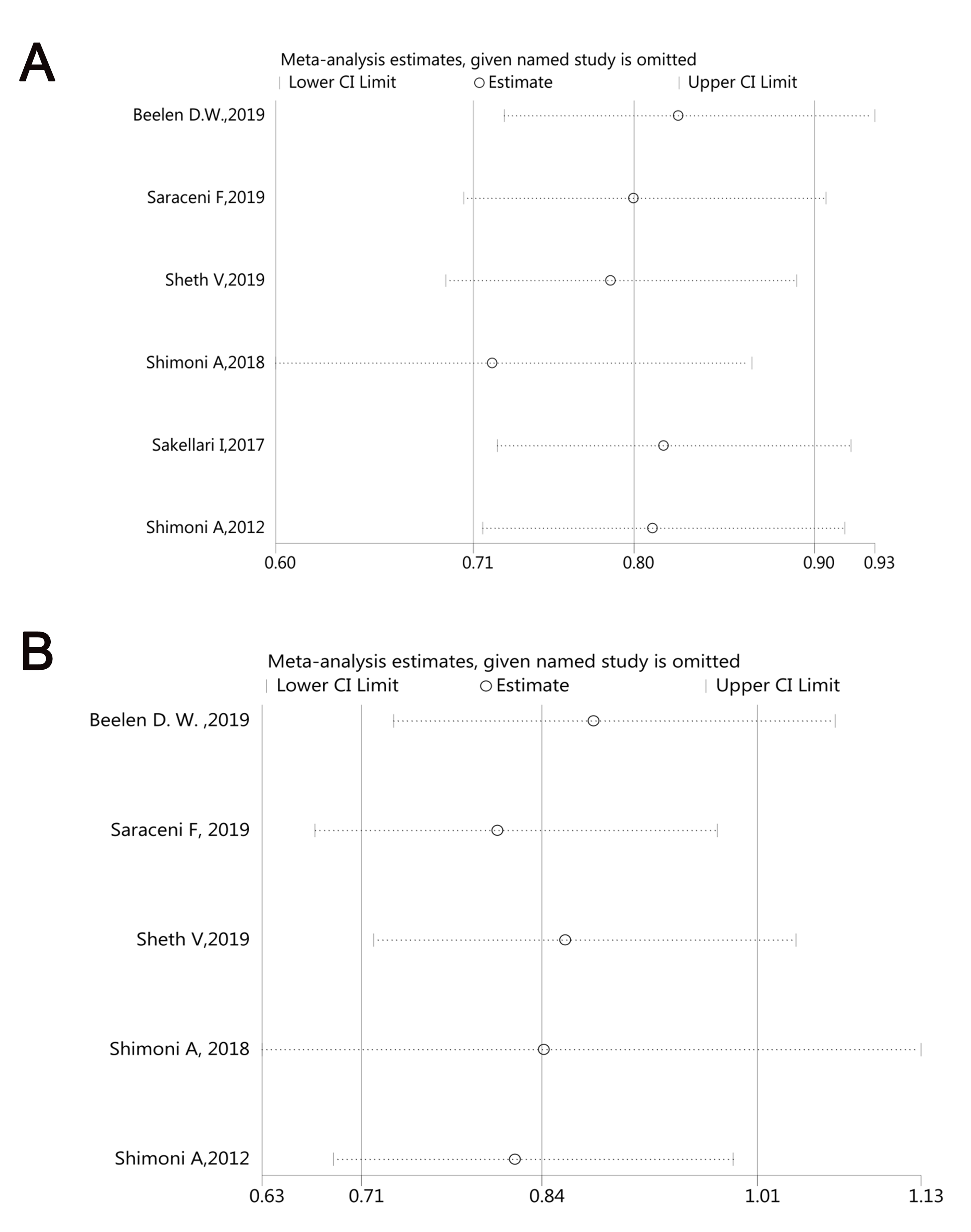

Supplement: Supplementary Figure 1 — (A) Sensitivity analysis of overall survival. (B) Sensitivity analysis of non-relapse mortality [file Image_1.tif]

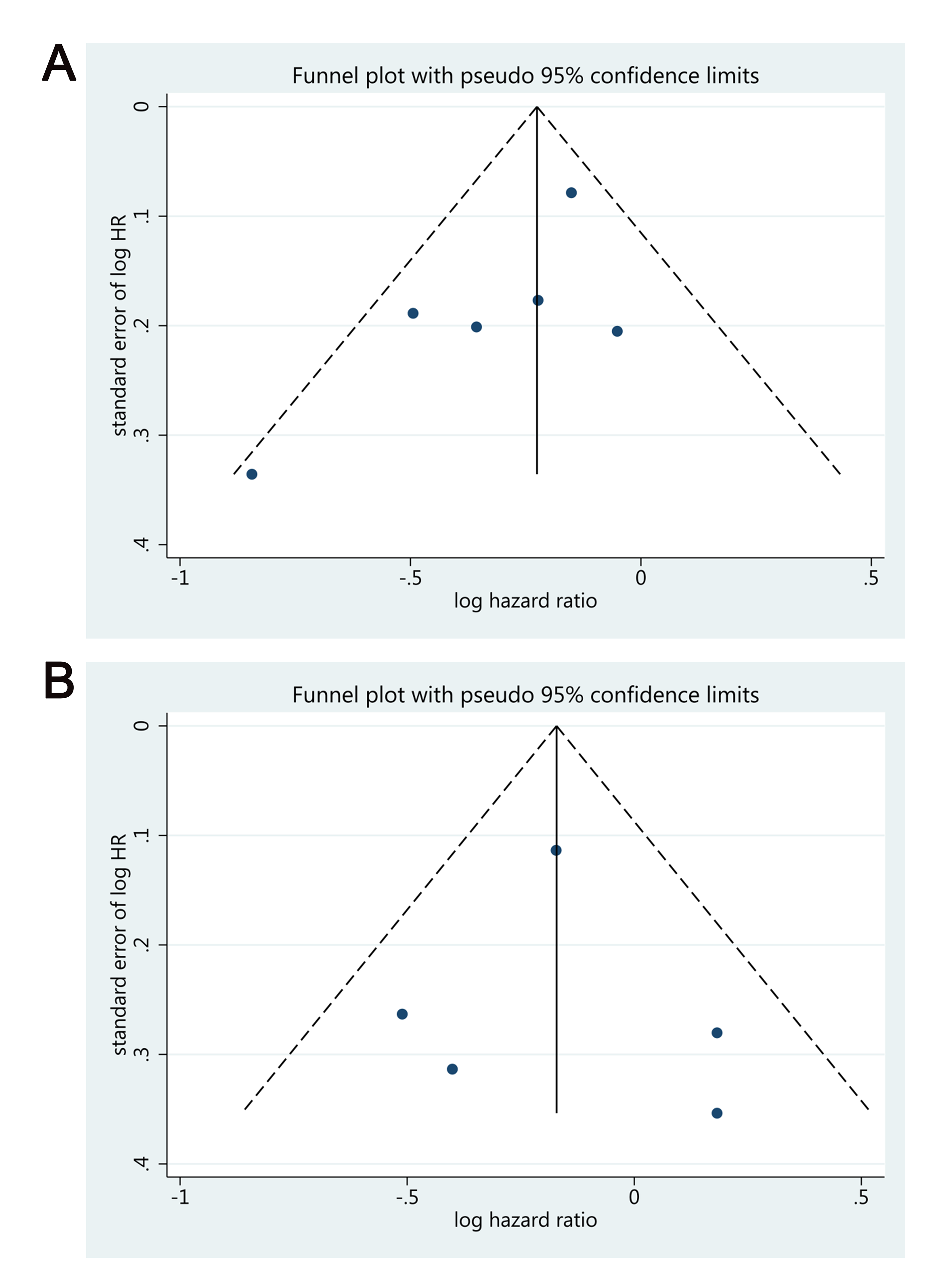

Supplement: Supplementary Figure 2 — (A) Funnel plot of overall survival. (B) Funnel plot of non-relapse mortality. [file Image_2.tif]
