# Supplementary material for: Long-Term Outcomes of Treosulfan- vs. Busulfan-Based Conditioning Regimen for Patients With Myelodysplastic Syndrome and Acute Myeloid Leukemia Before Hematopoietic Cell Transplantation: A Systematic Review and Meta-Analysis
Source: Front Oncol. 2020 Dec 16;10:591363. doi: 10.3389/fonc.2020.591363 (PMC7793760; doi:10.3389/fonc.2020.591363)
Supplement: Supplementary file 3 [file Table_1.docx]

Supplementary Table S1. Reasons for the exclusion of 20 studies

| **Study** | **Reasons for exclusion** |
| --- | --- |
| Gyurkocza 2014 [1] | Patients included age=1. |
| Michallet 2012 [2] | Patients included myeloid malignancies and lymphoid malignancies. |
| Hilgendorf 2011 [3] | Patients included non-Hodgkin’s lymphoma. |
| Shimoni 2005 [4] | Patients included multiple myeloma and non-Hodgkin’s lymphoma. |
| Steckel 2018 [5] | High-dose melphalan, followed by a total body irradiation (4×2 Gy)-based or a treosulfan-based dose-adapted conditioning therapy. |
| Deeg 2018 [6] | Compared treosulfan+ fludarabine+ TBI vs treosulfan+ fludarabine. Patients included age=2. |
| Shimoni 2017 [7] | Two treosulfan doses (total 36 or 42 g/m^2^) . |
| Nagler 2017 [8] | Compare three treosulfan doses (30, 36, or 42 g/m^2^). |
| Ruutu 2011 [9] | Group base on CTCAE grade, not on treosulfan. |
| Chemnitz 2011 [10] | Single arm TBI with treosulfan. |
| Shimoni 2007 [11] | Single arm ﬂudarabine 30 mg/m^2^ on days 76 to 72, and treosulfan 12 g/m^2^ on days 76 to 74. |
| Penack 2007 [12] | Various regimens and dosages. |
| Kröger 2006 [13] | Dose-reduced conditioning regimen consisting of treosulfan and fludarabine followed by aSCT in patients with secondary AML or MDS. |
| Casper 2004 [14] | Single arm treosulfan 10 g/m^2^ intravenously daily for 3 days and fludarabine 30 mg/m^2^ intravenously daily for 5 days. |
| Casper 2004 [15] | Single arm combining 3×10 g/m^2^ of treosulfan with 5×30 mg/m^2^ of fludarabine. Patients included various hematological malignancies. |
| Ram 2019 [16] | No available data reported. |
| Holtick 2017 [17] | No available data reported. |
| Fein 2018 [18] | The impact of individual comorbidities on non-relapse mortality not treosulfan. |
| Shelikhova 2019 [19] | Language is Russian. |
| Baǐdil'dina 2010 [20] | Language is Russian. |

1. Gyurkocza, B., et al., *Treosulfan, fludarabine, and 2-Gy total body irradiation followed by allogeneic hematopoietic cell transplantation in patients with myelodysplastic syndrome and acute myeloid leukemia.* Biology of Blood and Marrow Transplantation, 2014. **20**(4): p. 549-555.

2. Michallet, M., et al., *Phase II prospective study of treosulfan-based reduced-intensity conditioning in allogeneic HSCT for hematological malignancies from 10/10 HLA-identical unrelated donor.* Annals of Hematology, 2012. **91**(8): p. 1289-1297.

3. Hilgendorf, I., et al., *Retrospective analysis of treosulfan-based conditioning in comparison with standard conditioning in patients with myelodysplastic syndrome.* Bone Marrow Transplantation, 2011. **46**(4): p. 502-509.

4. Shimoni, A., et al., *Hematopoietic stem-cell transplantation from unrelated donors in elderly patients (age>55 years) with hematologic malignancies: Older age is no longer a contraindication when using reduced intensity conditioning.* Leukemia, 2005. **19**(1): p. 7-12.

5. Steckel, N.K., et al., *High-dose melphalan-based sequential conditioning chemotherapy followed by allogeneic haematopoietic stem cell transplantation in adult patients with relapsed or refractory acute myeloid leukaemia.* British Journal of Haematology, 2018. **180**(6): p. 840-853.

6. Deeg, H.J., et al., *Transplant Conditioning with Treosulfan/Fludarabine with or without Total Body Irradiation: A Randomized Phase II Trial in Patients with Myelodysplastic Syndrome and Acute Myeloid Leukemia.* Biology of Blood and Marrow Transplantation, 2018. **24**(5): p. 956-963.

7. Shimoni, A., et al., *Missing HLA C group 1 ligand in patients with AML and MDS is associated with reduced risk of relapse and better survival after allogeneic stem cell transplantation with fludarabine and treosulfan reduced toxicity conditioning.* American Journal of Hematology, 2017. **92**(10): p. 1011-1019.

8. Nagler, A., et al., *Long-term outcome after a treosulfan-based conditioning regimen for patients with acute myeloid leukemia: A report from the Acute Leukemia Working Party of the European Society for Blood and Marrow Transplantation.* Cancer, 2017. **123**(14): p. 2671-2679.

9. Ruutu, T., et al., *Reduced-toxicity conditioning with treosulfan and fludarabine in allogeneic hematopoietic stem cell transplantation for myelodysplastic syndromes:Final results of an international prospective phase II trial.* Haematologica, 2011. **96**(9): p. 1344-1350.

10. Chemnitz, J.M., et al., *Intermediate intensity conditioning regimen containing FLAMSA, treosulfan, cyclophosphamide, and ATG for allogeneic stem cell transplantation in elderly patients with relapsed or high-risk acute myeloid leukemia.* Annals of Hematology, 2011: p. 1-9.

11. Shimoni, A., et al., *Fludarabine and treosulfan: A novel modified myeloablative regimen for allogeneic hematopoietic stem-cell transplantation with effective antileukemia activity in patients with acute myeloid leukemia and myelodysplastic syndromes.* Leukemia and Lymphoma, 2007. **48**(12): p. 2352-2359.

12. Penack, O., et al., *A novel method to quantify and characterize leukemia-reactive natural killer cells in patients undergoing allogeneic hematopoietic stem cell transplantation following conventional or reduced-dose conditioning.* International Journal of Hematology, 2007. **85**(4): p. 326-332.

13. Kröger, N., et al., *Reduced-toxicity conditioning with treosulfan, fludarabine and ATG as preparative regimen for allogeneic stem cell transplantation (alloSCT) in elderly patients with secondary acute myeloid leukemia (sAML) or myelodysplastic syndrome (MDS).* Bone marrow transplantation, 2006. **37**(4): p. 339-344.

14. Casper, J., et al., *Treosulfan and fludarabine: a new toxicity-reduced conditioning regimen for allogeneic hematopoietic stem cell transplantation.* Blood, 2004. **103**(2): p. 725-731.

15. Casper, J., et al., *Treosulfan/fludarabine: a new conditioning regimen in allogeneic transplantation.* Annals of hematology, 2004. **83 Suppl 1**: p. S70-71.

16. Ram, R., et al., *Sequential therapy for patients with primary refractory acute myeloid leukemia: A historical prospective analysis of the German and Israeli experience.* Haematologica, 2019. **104**(9): p. 1798-1803.

17. Holtick, U., et al., *Similar outcome after allogeneic stem cell transplantation with a modified FLAMSA conditioning protocol substituting 4 Gy TBI with treosulfan in an elderly population with high-risk AML.* Annals of Hematology, 2017. **96**(3): p. 479-487.

18. Fein, J.A., et al., *The impact of individual comorbidities on non-relapse mortality following allogeneic hematopoietic stem cell transplantation.* Leukemia, 2018. **32**(8): p. 1787-1794.

19. Shelikhova, L.N., et al., *αβ-T-cell-depleted haploidentical hematopoietic stem cell transplantation in children with chemorefractory acute myeloid leukemia.* Pediatric Hematology/Oncology and Immunopathology, 2019. **18**(2): p. 11-21.

20. Baǐdil'dina, D.D., et al., *[Recurrences of acute promyelocytic leukemia in children: experience with arsenic trioxide therapy and autologous hematopoietic cell transplantation].* Terapevticheskiǐ arkhiv, 2010. **82**(7): p. 20-25.
